# Dengue Virus-Induced Inflammation of the Endothelium and the Potential Roles of Sphingosine Kinase-1 and MicroRNAs

**DOI:** 10.1155/2015/509306

**Published:** 2015-11-02

**Authors:** Amanda L. Aloia, Alexander M. Abraham, Claudine S. Bonder, Stuart M. Pitson, Jillian M. Carr

**Affiliations:** ^1^Microbiology and Infectious Diseases, School of Medicine, Flinders University, Bedford Park, Adelaide, SA 5042, Australia; ^2^Centre for Cancer Biology, University of South Australia and SA Pathology, Adelaide, SA 5000, Australia

## Abstract

One of the main pathogenic effects of severe dengue virus (DENV) infection is a vascular leak syndrome. There are no available antivirals or specific DENV treatments and without hospital support severe DENV infection can be life-threatening. The cause of the vascular leakage is permeability changes in the endothelial cells lining the vasculature that are brought about by elevated vasoactive cytokine and chemokines induced following DENV infection. The source of these altered cytokine and chemokines is traditionally believed to be from DENV-infected cells such as monocyte/macrophages and dendritic cells. Herein we discuss the evidence for the endothelium as an additional contributor to inflammatory and innate responses during DENV infection which may affect endothelial cell function, in particular the ability to maintain vascular integrity. Furthermore, we hypothesise roles for two factors, sphingosine kinase-1 and microRNAs (miRNAs), with a focus on several candidate miRNAs, which are known to control normal vascular function and inflammatory responses. Both of these factors may be potential therapeutic targets to regulate inflammation of the endothelium during DENV infection.

## 1. Introduction

In response to tissue injury or infection the body initiates a series of cellular events, including (i) vasodilation, (ii) recruitment of neutrophils, (iii) production of vasoactive factors that will induce endothelial cell (EC) activation, contraction, and adhesion molecule expression, and (iv) production of chemotactic factors that will result in recruitment of monocyte/macrophages and lymphocytes from the circulation to the site of injury. The outcome of these processes is an induction of a localised inflammatory response that allows cells to move out of the vessels to the site of injury/infection to repair cellular damage and restrict the replication of a pathogen [1]. During a dengue virus (DENV) infection, a globally significant mosquito borne human pathogen with an estimated 50–100 million infections per annum, this normal process has gone awry [2–6]. DENV infection can be associated with excessive or prolonged inflammatory responses and EC contraction, permeability, and adhesion changes of the endothelium [7–11]. These vascular effects of DENV infection produce a spectrum of severity of disease with the most severe effects occurring late in infection during the time of defervescence and decline in viremia [12]. DENV effects on the vasculature can lead to fluid, protein, and blood loss from the vessels and bleeding from mucosal surfaces and sites such as the gastrointestinal tract [12]. In severe cases, DENV-induced vascular leakage can be life-threatening and lead to outcomes such as hypovolemic shock (dengue shock syndrome, DSS) or haemoconcentration and disseminated intravascular coagulation [4, 9, 12]. There are no antiviral therapies for DENV and there are only supportive measures to treat this vascular leak syndrome [13, 14].

While it is clear that the site of severe DENV-induced pathology is at the vasculature, the contribution of EC to this pathogenic process is still unclear. In this review we will discuss the production of inflammatory and innate factors, including from the EC themselves, during DENV infection. Moreover, we consider two factors, sphingosine kinase-1 (SK1) and microRNAs (miRNAs), which are both key to EC function, vascular integrity, and inflammation, as potential therapeutic targets to modify the inflammatory response in the endothelium and alleviate severe DENV pathology.

## 2. DENV Infection Is Associated with Altered Production of Inflammatory and Vasoactive Factors and Functional Changes to the Endothelium

The lack of widespread damage observed in the endothelium of postmortem DENV patient tissues, in combination with the ability of patients suffering from severe DENV to make a rapid and complete recovery, suggests that the loss of vascular integrity and function during DENV infection* in vivo* is not due to major damage to the endothelium. Instead, these effects are most likely a result of vasoactive factors released from DENV-infected cells [2, 9, 11]. Cells contributing to these altered vasoactive factors during DENV infection are traditionally thought to be circulating monocytes, tissue macrophages, or dendritic cells (DCs). Monocytes/macrophages and DCs are also major targets for DENV replication* in vivo* [15–18]. The role of these DENV-infected cells as sources of cytokines/chemokines and vasoactive factors is supported by studies showing altered production of factors such as interleukin- (IL-) 1, IL-6, macrophage inhibitory factor (MIF), TNF-*α*, and metalloproteinases from macrophages [19–24] or DC infected with DENV* in vitro* [20, 25–27]. Using* in vitro* models of EC barrier function based on movement of labelled macromolecules or changes in cell electrical resistance, soluble factors released from DENV-infected macrophages as well as direct infection of EC themselves (see Section 3 below) have been shown to induce permeability changes of an EC monolayer without any associated viral-induced cytopathic effect [19, 28–31]. In addition to factors that induce greater permeability changes, responses to infection such as production of type I interferon could also protect or help maintain vascular integrity [32]. Additionally, DENV infection of EC and endothelial permeability changes are also associated with alteration of cell surface molecules on EC such as the upregulation of EC adhesion molecules (VCAM-1 and ICAM-1) [28, 33] and VEGFR2 [34]. Soluble VCAM-1 is also reported to circulate at higher levels in DENV patients [35] and VEGF is elevated in the circulation of patients with more severe DENV infection [34, 36]. DENV infection of mice deficient in platelet activating factor receptor (PAFR), a receptor through which PAF can induce EC permeability changes, resulted in a reduction in DENV-induced disease without affecting viremia [37]. This suggests that protection against the DENV-induced effects on the endothelium can be afforded by reducing the responsiveness of EC to factors, such as PAF [37]. An interesting set of experiments by Puerta-Guardo et al. investigated EC function in the context of DENV infection of differentiated monocytic cells in the presence of a monoclonal antibody to DENV protein E [38]. Monocytic cells infected with DENV in the presence of the antibody produced significantly higher levels of the inflammatory cytokines TNF-*α* and IL-6 than cells infected in the absence of antibody. Exposure of EC monolayers to conditioned medium from the monocytes (infected with DENV in the presence of antibody) leads to rapid degradation in EC barrier function that was shown to be mediated by TNF-*α*, IL-6, and IL-12p70. Additionally, injection of the conditioned medium into mice resulted in increased vascular permeability in the lungs and sera, supporting the notion that factors released from DENV-infected monocyte/macrophages have a clear role in inducing the deleterious effects of DENV on the vasculature and suggesting that antibody-dependent enhancement plays a role in severe dengue infection pathologies.

In addition to macrophages and DC, other cell types such as mast cells [39, 40] and platelets [41] can produce vasoactive factors such as VEGF which influence EC activation and function. Furthermore, microarray analysis of peripheral blood mononuclear cells (PBMCs) from DENV-infected patients indicates upregulation of transcripts from interferon-stimulated and neutrophil-associated pathways and a correlation of these transcriptional changes with disease severity [42].

Levels of factors such as VEGF [34, 36], IL-6, IL-8, IFN-*β* [43, 44], and TNF-*α* [33, 45, 46] in the circulation of DENV-infected patients are positively associated with disease severity. Interestingly, elevated MIP-1*β* and IL-12 were associated with improved disease outcomes [47] and similarly higher levels of IL-12 are seen in patients with mild but not severe disease [48].

Taken together, these studies demonstrate that there are changes in the production of many factors released from the different cell types that are altered during a DENV infection and that these probably act in concert to induce inflammation and functional changes in the endothelium.

## 3. Endothelial Cells Are a Source of Inflammatory and Vasoactive Responses during DENV Infection

In addition to the production of factors cited above, altered production of cytokine, chemokine, and vasoactive factors by EC themselves may also arise from the DENV infection. Numerous studies using cell lines and primary EC sources have shown that EC are targets for DENV infection* in vitro* [32, 44, 49–57]. Analysis of postmortem tissue from human DENV infection, however, does not support a generalised DENV EC infection, although DENV replication has been identified in some EC [58–60]. DENV-infected EC* in vitro* show altered production of factors such as IL-8 and complement [50], IL-6, and tissue plasminogen activator (TPA) [31, 43, 44]. mRNA expression analyses in EC challenged with DENV have shown induction of a number of pathways in response to infection, including components of the dsRNA pathway/interferon response, such as OAS and MxA, and inflammation, such as TNF-*α* [56, 61]. Microarray analysis of DENV-infected human umbilical vein EC (HUVEC), in a model where more than 80% of the cells are infected, has demonstrated large increases in expression of cytokines (including CXCL9/10/11 and IL-7), complement factors, IFN-*β*, and IFN-inducible genes such as viperin at 24 hours after infection [62]. In this study, the expression of inflammatory cytokines and chemokines was demonstrated to be dependent on IFN-*β* and not IFN-*α* release from EC [62]. Our own studies have similarly shown IFN-*β* dependent induction of innate antiviral responses via induction of mRNA for interferon stimulated genes (ISG) such as viperin, OAS1, and IFIT-1 in DENV-infected EC [55]. Other studies have demonstrated RIG-I dependent induction of interferon and inflammatory cytokines, including IFN-*β*, IL-6, IL-8, and RANTES, from DENV-infected EC [53]. Knockdown of RIG-I expression did not affect DENV replication, prompting the authors to suggest that RIG-I dependent cytokine and adhesion molecule expression contributes to DENV pathogenesis through promotion of the inflammation response (via leukocyte recruitment to EC). In our DENV EC infection model, using primary HUVEC, only a small proportion of EC become infected, but this has major consequences for the ability of EC to maintain normal barrier function* in vitro*, particularly following exposure to TNF-*α* [55]. These* in vitro* studies demonstrate that EC can become DENV-infected resulting in type 1 interferon responses and production of inflammatory factors that have vasoactive properties. Production of these factors from the EC themselves may act in an autocrine/paracrine manner to have larger and measurable functional changes on the endothelium as a whole.

Important roles for EC in viral induced inflammatory responses are also supported by mouse models of infection. In a mouse model of dengue haemorrhage, DENV inoculation resulted in DENV-infected EC, increased TNF-*α* production, macrophage recruitment, and EC apoptosis [63, 64]. In this model, TNF-*α* production originated from infiltrating macrophages. EC in the haemorrhage tissues were DENV-infected and demonstrated TNF-*α*-enhanced production of reactive nitrogen and oxygen species, contributors to EC apoptosis and EC barrier damage. A study by Zellweger et al. also demonstrated that a mouse model of antibody-dependent enhancement of DENV infection causes a more severe DENV disease and is also associated with DENV infection of EC in the liver [65]. Mouse models for infections with other viruses have also shown EC to have a key role in inflammation. Studies of influenza A virus (IAV) have also shown that the respiratory endothelium is key to the inflammatory response and induction of cell infiltration in a mouse model of highly pathogenic IAV [66]. This response was mediated by sphingosine-1-phosphate (S1P) receptor 1 (S1PR1) on EC. Agonism of S1PR1 on EC suppressed cytokine and chemokine production, including IFN-*α*, TNF-*α*, and IL-6. In a primate model of hantavirus pneumonia, the respiratory endothelium is the main site for host immune responses to infection also suggesting broader roles for EC during inflammatory responses to viral infections [67]. While, as described above, postmortem analysis of human DENV-infected tissues has shown some evidence of DENV infection of EC, analysis of the inflammatory response in these tissue samples has not been addressed but may yield insight into similar roles of the endothelium in inflammation during DENV infection* in vivo*.

Thus, traditionally, it has been considered that monocyte/macrophages and DCs are the main contributors to altered cytokines/chemokines and vasoactive factors that affect the endothelium during DENV infection, but growing evidence suggests that the EC themselves may also play an important role in propagating this dysregulated proinflammatory process.

## 4. SK1 Is a Key Regulator of Vascular Integrity and Inflammation and Is Altered during DENV Infection

Sphingosine kinase is a cellular lipid kinase that phosphorylates the lipid sphingosine to form S1P. One important role of SK1 is in control of the cell survival-death balance or rheostat [68]. Additionally, SK1 and S1P have vital functions in maintenance of EC permeability and vascular integrity [69] as well as EC functions including modulation of cell adhesion molecule expression [70] and adhesion-mediated cell survival [71]. SK1 has also been described as an important intracellular component of cell signaling pathways [72], in particular the pathway for TNF-*α* stimulated activation of NF*κ*B [73, 74], a vital transcription factor for induction of many inflammatory factors [75]. The roles of SK1 and S1P in inflammation have been recently reviewed [76, 77]. Our studies have shown that SK1 is reduced late in DENV infection in a variety of different cell lines, leading to reduced TNF-*α* stimulated NF*κ*B activation [78, 79]. In contrast, early in DENV infection of EC, SK1 activity is increased and this is associated with enhanced TNF-*α* induced permeability of EC [55], consistent with reported sensitization of EC to TNF-*α* by overexpression of SK1 [80]. SK1 is also required for IL-1 stimulated IRF1 mediated production of chemokines such as CXCL10 and IL-6 [81]. Both TNF-*α* and IL-1 are prototype stimuli for type II activation of EC, resulting in NF*κ*B activation, production of inflammatory factors, increased adhesion molecule expression, and increased permeability of EC [1]. Thus, SK1 can be altered by DENV infection in EC and this will have consequences for responses to inflammatory and vasoactive mediators such as TNF-*α* and maintenance of vascular integrity.

### 4.1. Can We Target SK1 Activity in the Endothelium to Reduce DENV-Induced Permeability of the Vasculature?

**Figure 1:**
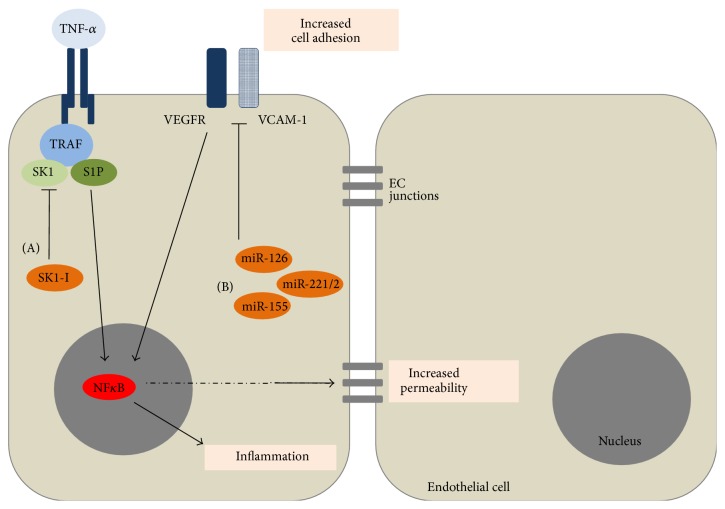
TNF-*α* or VEGF-mediated NF*κ*B activation promotes inflammation and opening up of EC junctions leading to increased vascular permeability. Increased VCAM-1 is associated with altered EC function and recruitment of immune cells. We propose (A) Inhibition of SK1. (B) Increased miRNA levels will reduce TNF-*α* or VEGF-mediated NF*κ*B activation and reduce VCAM-1 expression leading to maintenance or improvement of vascular integrity.

As stated above, growing evidence suggests a positive association between SK1 activity and TNF-*α* responses. Additionally, reports show that TNF-*α* is strongly linked to DENV disease in mice [63, 64]. TNF-*α* antibody can reduce the DENV-induced lethality in mice [82] and high levels of TNF-*α* in the circulation correlate with disease severity in humans [45, 46]. Therefore, inhibiting SK1 activity may prevent the pathogenic effects of TNF-*α* actions on the EC during DENV infection, as outlined in Figure 1. SK inhibitors are in development as anticancer therapeutics [83, 84] and perhaps these could be investigated for DENV treatment. Although this would require a fine therapeutic balance between inhibition of the deleterious role for SK1 in promoting inflammatory responses, whilst maintaining essential roles of SK1 in maintaining vascular integrity, the use of SK1 inhibitors has been shown to inhibit the induction of EC permeability by thrombin and neutrophils in an* in vitro* model and thus this selectivity of action is feasible [85]. Furthermore, recent advances in the selective targeting of inhibitors specifically to cytokine-activated EC have shown success in reducing NF*κ*B activation* in vivo* [86]. This suggests that a similar approach may be possible for targeting SK1 in these cells during the activation of EC associated with DENV infection.

S1P can act as a bioactive sphingolipid in signaling pathways inside cells or can be secreted from cells and act on a family of S1P receptors (S1P_1–5_) [87, 88]. S1P levels have recently been reported to be reduced in the circulation of DENV patients and thus may influence vascular integrity [89]. A number of agents are currently in development to modulate the SK/S1P axis by agonising or antagonising specific S1P receptors [90–92]. As discussed earlier, agonism of S1P receptors in EC suppresses cytokine and chemokine production [66], while S1P itself has been shown to alleviate the EC permeability induced by Hantavirus infection* in vitro* [93]. FTY720 is an agent that modulates all S1P receptors, except S1P receptor-2, and is already in clinical use for multiple sclerosis (MS) due to its lymphopenia-inducing properties [94]. FTY720 can block TNF-*α* induced EC permeability* in vitro*. Indeed, through its agonism of S1PR1, FTY720-mediated increased endothelial barrier function has been proposed as one mechanism whereby this agent inhibits lymphocyte egress from lymph tissue [95, 96]. Similarly, use of S1PR1 agonists such as FTY720 could be of benefit to protect the vasculature against the TNF-*α* mediated induction of EC permeability during DENV infection.

## 5. MicroRNAs: An Additional Mediator of EC Inflammation during DENV Infection?

First discovered in 1993, miRNAs are a class of noncoding RNA with a length of around 22 nucleotides [97]. The expression, processing, and specific mechanisms by which this highly conserved and important class of molecules functions has been well reviewed elsewhere [98]. Through mRNA binding and subsequent inhibition of translation and/or induction of mRNA degradation, miRNAs fine-tune the expression of cellular genes making them key regulators of numerous cellular processes. It is estimated that at least 60% of protein coding genes have miRNA target sites in the 3′-untranslated regions (UTRs) of their encoded mRNAs and a single miRNA can regulate translation of hundreds of genes [99]. miRNAs have well established roles in endothelial cell inflammation but substantially less is known about the relationship between miRNAs and DENV-induced EC inflammation and reduced vascular integrity or, indeed, about miRNAs and DENV replication itself. To date there is little knowledge regarding modification of miRNA expression in DENV-infected EC and the possibility of miRNA transfer, for example, through exosomes, between DENV-infected monocytes/macrophages and EC has not been investigated. Additionally, the inflammatory stimuli, such as IFNs, TLR ligands, and chemokines, secreted from DENV-infected monocytes, macrophages, and DC, would certainly induce changes in miRNA levels in EC but this also has not been specifically investigated in the context of DENV infection, for example, through the use of cell coculture techniques or application of supernatant from DENV-infected immune cells to cultured EC.

DENV infection, however, has been shown to modulate the abundance of a variety of miRNAs in nonendothelial cells. Although not specifically the subject of this review, these studies highlight the connection between DENV replication and miRNAs and, furthermore, changes in miRNA abundance in these cells contribute to the release of inflammatory stimuli, which would subsequently influence EC inflammation.

### 5.1. DENV Infection and miRNA Expression Changes in Non-EC Sources

Microarray analysis of miRNA levels in whole blood of patients at various stages after DENV infection (up to 14 days after infection) compared to uninfected patients detected a total of 348 miRNAs which were differentially expressed [100]. Groups of miRNAs were found to be specifically differentially regulated at the various stages of infection presenting the possibility that some of these miRNAs may be useful as biomarkers of infection progression or of DENV infection itself. Indeed, 3 miRNAs, miR-24-1-5p, miR-512-5p, and miR-4640-3p, were found to be differentially regulated following DENV but not IAV infection. Of the many differentially regulated miRNAs in DENV-infected patients, none appear to have well-known roles in EC inflammation or barrier integrity, although miR-299-3p which was upregulated in all DENV-positive patients is known to play a role in EC senescence [101].

Changes in the abundance of miRNAs in DENV-infected PBMCs have also been observed by microarray analysis of infected cells [102]. Qi et al. observed significant changes in 19 miRNAs with the most significant change in expression levels observed for miR-4290, miR-1290, miR-33a, and miR-let-7e (upregulated) and miR-106b, miR-20a, and miR-30b (downregulated). Target prediction suggested that miR-let-7e may target the UTR of IL-6 and CCL3, while miR-451 and miR-106b could potentially target MIF and CCL5, and miR-4279 could potentially target CXCL1. Functional demonstration of these mRNAs as miRNA targets was not conducted, however, nor has it been reported elsewhere. A specific investigation into the role of these miRNA alterations in DENV-induced changes in PBMCs, in particular if they have any relationship with the chemokine release (especially CCL5, IL-6, and IL-8) that was observed from DENV-infected PBMCs in this same study, has not been examined [102]. The majority of the miRNAs differentially regulated in DENV-infected PBMCs have been implicated in other cellular processes and disease states including cancer (e.g., miR-1290), mitochondrial function (e.g., miR-33a), and apoptosis (miR-106b) and may be related to similar changes in cell growth during a DENV infection [103–105].

Zhu et al. specifically investigated the role a of a single miRNA, miR-30e^*∗*^, in DENV-1, -2, or -3 infection of HeLa cells [106]. Following DENV infection, increased levels of miR-30e^*∗*^ were observed. Furthermore, U937, HeLa, or PBMC cells transfected with miR-30e^*∗*^ demonstrated increased IFN-*β*, OAS1, MxA, and IFITM1 production (compared to cells transfected with a negative control mimic). Induction of IFN-*β* appeared to occur through a direct interaction of miR-30e^*∗*^ with the 3′-UTR of I*κβα* resulting in a decrease in cellular levels of I*κβα*. Reduced levels of I*κβα* would subsequently lead to activation of NF*κ*B and induction of IFN-*β*. Thus when U937, HeLa, and PBMC cells were transfected with miR-30e^*∗*^, DENV-2 replication was inhibited. Potentially, targeted delivery of miR-30e^*∗*^ to PBMC could represent a therapeutic strategy for inhibiting DENV infection.

A broader role for DENV infection in miRNA modulation was observed by Kakumani et al. who demonstrated that DENV infection of Huh7 cells resulted in a decrease in the mRNA levels of miRNA processing proteins, including Dicer, and a subsequent downregulation of 143 of the 151 miRNAs randomly investigated [107]. The inhibition of miRNA production was, at least in part, mediated by the middle and C-terminal domains of the DENV protein, NS4B, suggesting a direct ability of DENV to modulate the cellular proteins involved in miRNA synthesis.

In contrast to these studies, a general role for miRNAs in regulating virus replication, including DENV, has been questioned [108]. Knockout of the miRNA processing protein Dicer from 293T cells, which renders them incapable of producing mature miRNA (or siRNA), did not significantly alter the replication of 10 different test viruses. Interestingly, however, of the 10 viruses investigated in this study, DENV was the only one to show a reproducible lower level of replication in the Dicer-negative cells, which could not be attributed to slower cell growth.

Overall, however, there is an abundance of evidence demonstrating that DENV infection modulated miRNA levels in a variety of cell types. No studies were conducted in EC however, and thus any relationship that miRNAs identified above have with DENV-induced EC inflammation or barrier integrity would be indirect. For example, in the studies of Jong et al. and Qi et al. in blood or PBMC, the analysed samples would not contain any intracellular EC-associated miRNAs. Any potential contribution of EC-derived miRNA to the circulation could only have arisen from miRNAs secreted from the EC (e.g., in exosomes) or from circulating EC, which have been observed in patients with severe DENV disease [35]. In relation to the circulating changes in miRNAs that have been observed, if the upregulation of miR-let-7e documented by Qi et al., for instance, did indeed modulate PBMC production of IL-6, this may subsequently promote EC inflammation [102]. In particular, it is interesting to speculate on a role for miR-30e^*∗*^, which, as discussed, can influence interferon responses, in DENV-induced modulation of EC function as well as DENV infection of EC. Furthermore, we and others have demonstrated that IFN-*β* is induced in DENV-infected EC [8, 55]. It would be interesting to investigate the role of miR-30e^*∗*^ in both EC function and interferon responses either following direct infection of EC or in EC that have been exposed to, for example, supernatant from DENV-infected PBMC or macrophages.

### 5.2. miRNAs That May Contribute to EC Inflammation and Barrier Integrity during DENV Infection

The role of miRNAs in EC inflammation and barrier integrity is very well established and there are several excellent reviews on the topic [109–111]. However, there has been little investigation into changes in EC miRNA levels during DENV infection. Here we will focus on a few miRNAs that may be of particular interest, given their well-established role in EC inflammation and barrier integrity in other physiological states. Inflammatory stimuli such a IFNs, lysophosphatidic acid (LPA), ILs, TNFs, and TLR ligands, which are induced by DENV infection, are known to alter expression of several miRNAs in EC including, but not limited to, miR-223, miR-126, miR-155, and miR-221/222 [109–111]. Here we will discuss the known functions of these miRNAs in EC function and their potential role in DENV infection.

#### 5.2.1. miR-223

Recently, DENV-induced regulation of a miRNA in EC was in fact demonstrated, although not in the context of EC inflammation and barrier integrity [112]. Detailed work by Wu et al. demonstrated that miR-223 was downregulated in EAhy926 cells (cultured human vascular endothelial cells) following DENV infection, while EAhy926 cells transfected with a miR-223 expression vector had reduced DENV replication [112]. Further analysis, based on target prediction of miR-223, showed that miR-223 inhibits translation of the microtubule destabilizer, stathmin-1. Stathmin-1 was shown to be upregulated following DENV infection of EAhy926, while inhibition of stathmin-1 expression had a negative effect on DENV replication. Thus, DENV infection of EAhy926 cells decreased expression of miR-223, resulting in an increase in stathmin-1 transcription and protein levels, which assists DENV replication through an as yet unknown mechanism. While the miR-223/stathmin-1 link described in the study of Wu et al. does not specifically link to any particular EC state, it does link DENV replication to miRNA function. Furthermore, miR-223 does have well-established roles in inflammation in the context of other cell types, in particular adipocytes (reviewed in [113]). In this context, miR-223 downregulation is associated with increased inflammation and thus it is plausible that miR-223 downregulation in DENV-infected EAhy926 cells may similarly contribute to induction of proinflammatory stimuli in these cells [113]. This is a new and developing area of EC biology and, of note, high EC levels of miR-223* in vitro* have been only recently reported likely due to the fact that miR-223 levels rapidly decreased when primary cells were cultured [114]. Interestingly, miR-223 can be transferred in microvesicles from activated platelets to EC, suggesting that circulating cells could modulate EC function by transfer of miR-223 [115].

#### 5.2.2. miR-126

**Figure 2:**
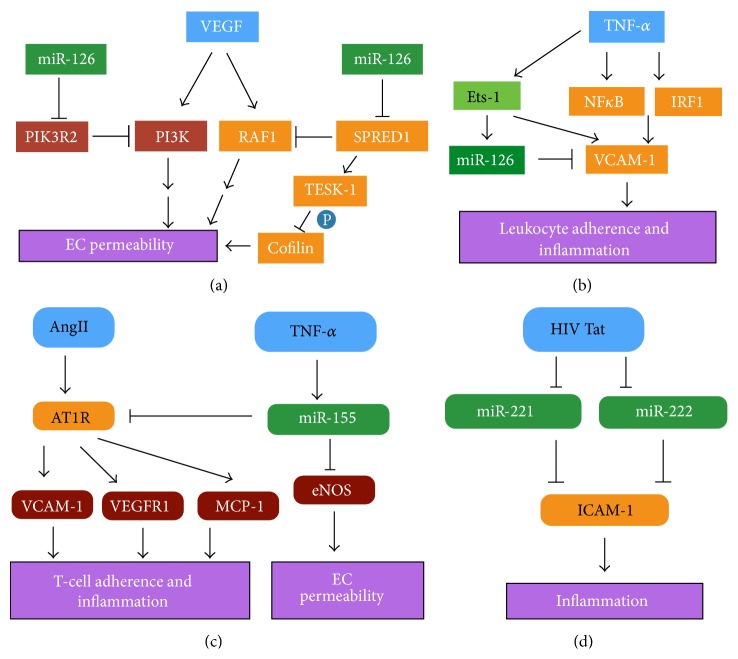
Roles of miR-126, miR-155, miR-221, and miR-222 in endothelial cell inflammation and maintenance of vascular integrity. (a) miR-126 regulates EC permeability through inhibition of PI3K and MAPK signaling pathways. miR-126 regulation of SPRED1 also indirectly regulates phosphorylation of cofilin and protein involved in maintenance of EC junctions. (b) Increased miR-126 levels result in a decrease in VCAM-1 levels, providing an anti-inflammatory effect. (c) miR-155 inhibits eNOS synthesis and thereby regulates EC permeability. Additionally, miR-155 inhibits Angiotensin-1 Receptor (AT1R) signaling, decreasing the expression of proinflammatory factors in EC. (d) HIV protein Tat can reduce transcription of miR-221 and miR-222, releasing their blockade on ICAM-1 translation and resulting in an inflammatory response.

**Figure 3:**
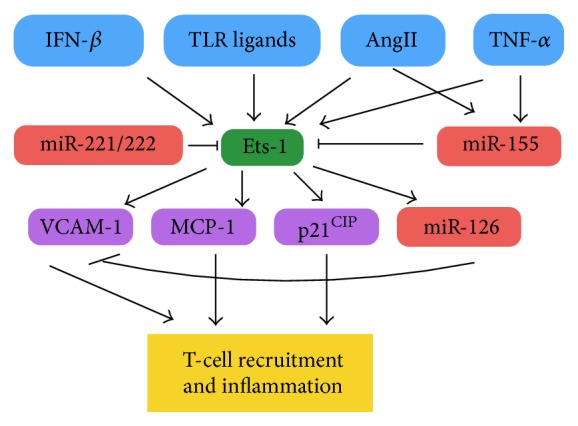
The interaction of miR-155, miR-221/222, and miR-126 in regulation of Ets-1 driven endothelial cell inflammation. A variety of proinflammatory extracellular factors induce Ets-1 transcription which subsequently induces expression of EC proteins involved in T-cell recruitment. miR-155 and miR-221/222 act to reduce translation of Ets-1, while miR-126 acts further downstream to reduce VCAM-1 translation.

miR-126 is one of the most abundantly expressed miRNAs in EC and is known to be involved in the inflammatory response, angiogenesis, and vascular integrity [116–118]. Expression of miR-126 is regulated by the transcription factor E26 transformation-specific sequence-1 and -2 (Ets-1 and Ets-2, further discussed below) [119]. Both Ets-1 and Ets-2^(−/−)^ and miR-126^(−/−)^ mice show a similar phenotype of impaired vascular formation and partial lethality [117, 118, 120, 121]. miR-126 represses the translation or degrades the mRNA of three key EC genes involved in vascular integrity and inflammation: SPRED1, PI3KR2, and VCAM-1 [117, 118, 122]. SPRED1 is a negative regulator of the MAP kinase pathway and interacts with testicular protein kinase-1 (TESK-1) to prevent the phosphorylation of cofilin [117, 118, 123, 124]. Unphosphorylated cofilin leads to disrupted adherens junctions and contributes to an increase in EC permeability. Overexpression of SPRED1 or knockdown of miR-126 also decreases responsiveness to VEGF, a factor crucial in maintaining EC permeability. Likewise, PI3KR2, a negative regulator of the PI3 kinase pathway, inhibits VEGF induced vascular integrity. The roles of miR-126 in EC permeability are summarised in Figures 2 and 3. miR-126 suppresses VCAM-1 levels and therefore is important in preventing initiation of inflammation in resting EC (Figure 3) [122]. Additionally, Ets-1, a transcriptional activator of miR-126, induces VCAM-1 expression in response to proinflammatory mediators. This coalesces as a neatly regulated negative feedback process by which VCAM-1 mediated inflammation can be controlled: Ets-1 induces VCAM-1 and miR-126, with miR-126 subsequently reducing transcription of VCAM-1 [119].

miR-126 also has a role in the innate responses of pDCs, with miR-126^(−/−)^ mice showing increased pDC apoptosis resulting in fewer circulating pDCs [125]. Those remaining pDCs have impaired function including reduced ability to produce IFN-*β* and IFN-*α* and to migrate to lymphoid tissues. Additionally, miR-126 was found to be a positive regulator of* Kdr*, the gene which encodes VEGFR2 in mice [125]. Therefore changes in the levels of miR-126 in DENV-infected pDCs are likely to contribute to EC inflammation through IFN-induced mechanisms.

#### 5.2.3. miR-155

A role for miR-155 in inflammation was first demonstrated by its upregulation in macrophages treated with either polyriboinosinic:polyribocytidylic acid (poly(I:C)) or IFN-*β* or TLR ligands [126]. miR-155 is abundant in EC and has been demonstrated to be crucial in TNF-*α* induced downregulation of endothelial nitric oxide synthase (eNOS), a regulator of EC permeability [127, 128]. In HUVEC cells, TNF-*α* was shown to induce miR-155 expression which subsequently bound to the 3′UTR of eNOS mRNA, inhibiting its translation. Sun et al. investigated this in relation to cardiovascular disease and, in this regard, reported that simvastatin treatment of HUVECs prevented TNF-*α* induced downregulation of eNOS and reduced TNF-*α* induced miR-155 expression. As eNOS (and NO it produces) is a regulator of EC permeability, it would be interesting to investigate miR-155 abundance and any subsequent changes in TNF-*α* induced eNOS expression in the context of DENV infection [129]. Interestingly, our own microarray analysis of DENV-infected murine embryonic fibroblasts (MEF) suggests that miR-155 is upregulated following DENV infection (Carr, unpublished observations). miR-155 has also been shown to reduce expression of the angiotensin-II (AngII) type-1 receptor (AT1R), resulting in a decrease in AngII-induced expression of VCAM-1, monocyte chemoattractant protein-1 (MCP-1), and vascular endothelial growth factor receptor (VEGFR1) and, ultimately, reducing T-cell recruitment [128]. These roles for miR-155 are summarized in Figure 2(c).

#### 5.2.4. miR-221/222

miRNAs miR-221 and miR-222 are often grouped together when studied (miR-221/222) due to the fact that their expression is likely to be simultaneously regulated and they are located close together on chromosome Xp11.3 [130]. miR-221 and to a lesser extent miR-222 are known to regulate ICAM-1 expression in EC in response to viral infection (Figure 2(d)) [131]. In HUVECs, the HIV protein Tat induces a downregulation in the expression of both miR-221 and miR-222, which leads to an increase in ICAM-1 expression and recruitment of monocytes. The reduction in both miR-221 and miR-222 levels and the increase in ICAM-1 were associated with activation of the NF*κ*B pathway. miR-221 has also been shown to downregulate expression of adiponectin receptor 1 (AdipoR1) in HUVECs, which subsequently inhibits NO production [132].

### 5.3. The Potential for Coregulation of miR-126, miR-155, and miR-221/222 during DENV Infection

Particularly interesting is the fact that miR-126, miR-155, and miR-221/222 share a regulation pathway involving Ets-1, a transcription factor that is important in EC inflammation [133, 134]. Transcription of Ets-1 itself can be induced by variety of proinflammatory stimuli including AngII, IL1-*β*, and TNF-*α* (reviewed in [134]). For example, in C57BL/6 mice, Ets-1 expression is initiated rapidly following activation of mouse aortic vascular smooth muscle and EC with AngII and leads to an increase in translation of MCP-1, the cyclin-dependent kinase inhibitor P21^CIP^, plasminogen activator inhibitor-1 (PAI-1), and VCAM-1 [133]. While MCP-1 and VCAM-1 contribute to monocyte recruitment to the EC, P21^CIP^ was shown to be associated with EC apoptosis [133]. PAI-1 inhibits the generation of proteins (e.g., plasmin) involved in remodeling of the extracellular matrix and thereby contributes to fibrosis. The link between miR-126, miR-155, miR-221/222, Ets-1, and the roles of these factors in EC function is complex but is simplistically summarised in Figure 3. Firstly, the transcription of miR-126 is positively regulated by Ets-1 which, in turn, is negatively regulated by miR-221/222 and miR-155. As discussed, induction of all of these factors occurs in the presence of proinflammatory stimuli including TNF-*α* (e.g., miR-155 and Ets-1), foreign antigens (e.g., miR-221/222), IFN-*β* (e.g., Ets-1 and miR-126), and AngII (e.g., miR-155). Subsequently the transcription of a variety of proinflammatory and EC regulatory mediators is further induced, including VCAM-1, MCP-1, and P21^CIP^. miR-155, miR-221/222, and miR-126 act to reduce the transcription of these proinflammatory stimuli.

Several of the proinflammatory mediators regulated by miR-126, miR-155, and miR-221/222 are known to be upregulated following DENV infection either in* in vitro* EC studies or in patient sera. MCP-1 is known to be highly expressed in patients with severe DENV disease (with hemorrhagic fever and shock syndrome), although the pathways leading to its high expression have not been investigated [135]. MCP-1 transcription can be induced by IL-1*β* and TNF-*α* and regulated by NF*κ*B and activator protein 1 (AP-1) or, as discussed above, in EC it can be induced by AngII and regulated by Ets-1 [133, 136]. It is interesting too to note that treatment of mice with the AngII type-1 receptor (AT1R) antagonist Losartan prior to DENV infection resulted in a decrease in DENV antigen presenting macrophages as well as a decrease in IL-1 production, compared to untreated, DENV-infected mice [137]. As discussed, soluble ICAM-1, soluble VCAM-1, and circulating EC are altered in the peripheral blood of patients with severe dengue infection [35].* In vitro*, exposure of EC to culture medium from DENV-infected monocytes results in a TNF-*α* mediated increase in VCAM-1 and ICAM-1 expression in the EC [28]. Although reports describing the relationship between DENV infection and serum VEGF levels have been conflicting, higher levels of soluble VEGFR1 are commonly reported in dengue-infected patients and may also be correlated with disease severity [28, 138].

Finally, there is precedent for regulation of miR-155, miR-126, and miR-222 in virus-infected EC. Andes hantavirus (ANDV), a virus that can also lead to hemorrhagic fever and vascular leakage* in vivo*, results in significant changes in the abundance of 23 miRNAs in infected HUVECs, including miR-155, miR-126, and miR-222 [139]. Following ANDV infection of HUVEC levels of miR-155 increased sixfold, while miR-222 levels increased threefold. Although miR-126 levels appeared to show a small (twofold) increase following ANDV infection, SPRED1 and PIK3R2 mRNA levels (see Section 5.2.2) were increased tenfold and sevenfold, respectively. This is unexpected given that miR-126 negatively regulates SPRED1 and PIK3R2 transcription and the authors concluded that ANDV interferes with miR-126 by an as yet unknown mechanism. Potentially too, the increase in miR-126 levels was not sufficient to regulate SPRED1 and PIK3R2 mRNAs, and indeed the abundance of miR-155 would act as a negative regulator of miR-126 transcription (Figure 3). It is likely that with more research more roles for these miRNAs in EC function during viral infection and inflammation will be uncovered to expand the complex interactions depicted in Figure 3.

From the proceeding discussion it can be seen that DENV modulation of EC miRNA levels is highly probable and likely to go beyond the only report so far of miRNA modulation in DENV-infected EC [112]. Whether miRNA modulation in EC is a result of DENV infection of the EC themselves or activation of EC by stimuli released from DENV-infected monocytes, macrophages or pDCs is, in some regards, irrelevant. One might predict a simple model whereby at time points early after DENV infection levels of, for example, the miRNAs discussed here may be reduced thereby releasing inhibition of the inflammatory response. In a resolved course of infection, restoration of normal EC miRNA levels would occur. In situations of prolonged reduction of these miRNA levels, we would also anticipate prolonged and increased levels of factors with known inflammatory and EC permeability increasing properties such as VCAM-1 and PI3K. Such an extended reduction of miRNA levels in EC that control inflammation and EC permeability may be an important contributor to the reduced vascular integrity and uncontrolled inflammation seen late in DENV infection with accompanying severe DENV disease. Potentially, direct infection of EC could contribute to decreased miRNA levels through sequestration of the miRNA, in a similar manner to HCV interaction with miR-122 [140]. Although this simple model is appealing, the mechanisms are likely more complex.

Importantly, if a role for miRNAs in DENV pathogenesis could be identified, it may present a target for antiviral miRNA enantiomers or delivery of miRNA mimics which may help to control the inflammation and vascular permeability that occurs in late-stage, severe DENV infection and limit postinfection progression of inflammation. There are several methods by which miRNAs can be delivered, including some which can specifically target EC [141–143] and the exciting potential to understand the role of miRNAs in EC function during DENV infection remains to be explored.

## 6. Summary and Perspective

Evidence demonstrates that severe DENV can be considered as an infectious trigger of an acute vascular disorder associated with an inflammatory process. The inflammatory process may be driven, at least in part, by responses of the endothelium itself to infection. There are currently no clinically available antiviral treatments to reduce DENV replication, and treatment of the DENV vascular leak syndrome is by supportive measures only. By understanding the factors driving the inflammatory process and responses of the EC to DENV, we have rationalized new potential therapeutic targets, SK1 and miR-126, miR-155, and miR-221/222, to treat the severe symptoms of DENV infection. A key factor for success of these potential therapies aimed at the process of inflammation may be the timing of administration of treatment. Vascular leak syndrome typically occurs late in infection, after viremia decline, and at this stage of infection it may be clinically safe to dampen down the inflammatory factors affecting the vasculature without the risk of exacerbating DENV infection via any immunosuppressive effects. Additionally, in accordance with WHO 2009 guidelines, patients now may be classified as DENV with or without warning signs, with the former at highest risk of development of severe DENV. These patients may be an important target group for therapy to modulate molecules in the EC and thus to ensure vascular integrity is maintained and attenuate the onset of life-threatening DENV-induced disease.
